# Hantavirus Cardiopulmonary Syndrome and Diffuse Alveolar Hemorrhage in the Era of COVID-19

**DOI:** 10.1155/2021/8800500

**Published:** 2021-09-24

**Authors:** Khizar Hamid, Swaminathan Perinkulam Sathyanarayanan, Touba Naim, Muhammad Hamza, Mirza Omer Mahmood Baig, Emad Abu Sitta

**Affiliations:** ^1^Internal Medicine, University of South Dakota Sanford School of Medicine, Sioux Falls, SD, USA; ^2^Infectious Disease, Sanford USD Medical Center, Sioux Falla, SD, USA

## Abstract

Hantavirus Cardiopulmonary Syndrome (HCPS) can occur after infection with Hantavirus which can occur by inhaling aerosolized rodent urine, feces, and saliva contaminated with the virus. It presents with the rapid development of pulmonary edema, respiratory failure, and cardiogenic shock with the hallmark being microvascular leakage. We report a patient with a history of alcohol abuse and recent exposure to mice and sick kittens who presented with cough with sputum production, shortness of breath, orthopnea, and new-onset lower extremity edema. Imaging revealed bilateral infiltrates more common on the left with an unremarkable echocardiogram. Testing for COVID-19, Human Immunodeficiency Virus (HIV), influenza, bacterial pneumonia including tuberculosis and methicillin-resistant *Staphylococcus aureus* (MRSA), aspergillosis, histoplasmosis, *Blastomyces,* and *Coccidiodes* was negative. Bronchoscopy and bronchoalveolar lavage revealed diffuse alveolar hemorrhage (DAH) and were negative for acid-fast bacilli and *Nocardia* cultures. He was further tested for Hantavirus, Q fever, leptospirosis, toxoplasmosis, and empiric treatment with doxycycline initiated. His Hantavirus IgM antibody came back positive. Human Hantavirus infection occurs after inhalation of infected rodent excreta; fortunately, human-to-human transmission has not been documented. HCPS most commonly occurs due to the Sin Nombre virus (SNV), has a case fatality rate of 50%, and is a notifiable disease in the United States. It has 3 distinct phases, prodromal, cardiopulmonary, and convalescent/recovery. The cardiopulmonary phase occurs from increased permeability of pulmonary capillaries and in severe cases can progress to cardiogenic shock. Diagnosis is based on the presence of IgM and IgG Hantavirus antibodies. Treatment is mainly supportive; however, patients are usually treated with broad-spectrum antibiotics while workup is underway. In animal models, ribavirin and favipiravir are only effective when administered in the prodromal phase. If suspicion of Hantavirus infection exists, early mobilization to the intensive care unit for treatment is recommended. Extracorporeal membrane oxygenation (ECMO) has been suggested to improve outcomes in severe HCPS with refractory shock.

## 1. Introduction

Hantaviruses are RNA viruses belonging to the Bunyaviridae family whose transmission in humans is caused by inhaling aerosolized rodent urine, feces, and saliva contaminated by the virus [1]. They mainly attack vascular endothelial cells along with alveolar macrophages and follicular dendritic cells. Another potential infection site is the epithelium of renal tubules [2]. Hemorrhagic fever with renal syndrome (HFRS) and Hantavirus Cardiopulmonary Syndrome (HCPS) are two syndromes caused by Hantavirus in humans [1, 2]. HCPS, also known as Hantavirus pulmonary syndrome, is a severe illness denoted by the rapid development of pulmonary edema, respiratory failure, and cardiogenic shock. The hallmark of HCPS is microvascular leakage [3]. Here, we report a 33-year-old man with HCPS presenting with diffuse alveolar hemorrhage (DAH).

## 2. Case Presentation

A 33-year-old Native American man, a welder by profession, with a past medical history significant for alcohol use, hypertension, and asthma presented to the hospital with complaints of cough with occasional bloody sputum production. His cough started four days before presentation, was initially dry but progressively worsened, and became productive. It was also associated with dyspnea and orthopnea along with new-onset lower extremity edema. He further endorsed preceding nausea, vomiting, and diarrhea without abdominal pain, melena, or hematemesis. He reported an extensive history of alcohol consumption and had quit smoking 6 years ago.

On physical examination, he appeared restless and his vitals comprised a temperature of 98.5°F, blood pressure 158/91 mm of Hg, respiratory rate 30 breaths/minute, pulse 116 beats/minute, oxygen saturation 96% on 2 liters of supplemental oxygen via a nasal cannula, weight 290 pounds, and a body mass index of 39.32. His pertinent laboratory findings included a white blood cell count of 16.3 K/uL, hemoglobin 8.6 g/dL, platelets 124,000 K/uL, potassium 3.0 mEq/L, sodium 125 mEq/L, magnesium 1.6 mg/dL, folate level 5.7 ng/mL, creatinine kinase 455 U/L, lactate dehydrogenase 310 U/L, albumin 2.9 g/dL, CRP 16.9 mg/L, lactic acid 3.1 mmol/L, procalcitonin 0.14 ng/mL, and D-dimer 10.56 ug/mL FEU. His ethanol level was 96.7 mg/dL, while liver function tests showed aspartate transaminase 132 U/L, alanine transaminase 33 U/L, and alkaline phosphatase 219 U/L. Testing for SARS-CoV-2, Human Immunodeficiency Virus (HIV), influenza, and methicillin-resistant *Staphylococcus aureus* (MRSA) nasal screen was negative.

**Figure 1:**
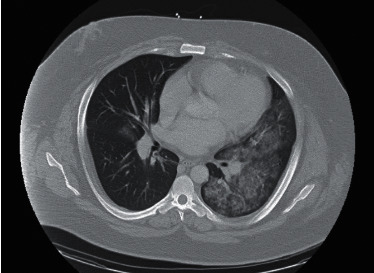
Computed tomographic angiogram of the chest showing bilateral infiltrates more extensive on the left.

His computed tomographic angiogram of the chest (Figure 1) revealed bilateral infiltrates more extensive on the left as compared to the right and excluded pulmonary embolism. Ultrasound abdomen revealed hepatomegaly. An echocardiogram showed an ejection fraction of 65–70% with moderate left ventricular hypertrophy, left atrial enlargement, and right atrial dilation. He was started on empiric treatment with ceftriaxone 2 gm daily and doxycycline 100 mg twice a day for suspected bacterial pneumonia along with management of possible alcoholic hepatitis with thiamine and folic acid and Clinical Institute Withdrawal Assessment for Alcohol (CIWA) protocol.

**Figure 2:**
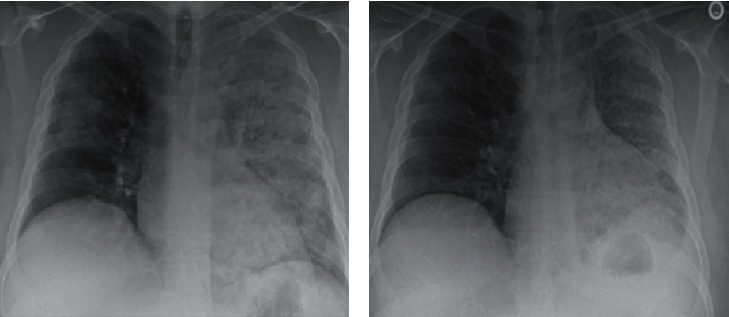
(a) Chest X-ray showing worsening left-sided infiltrates. (b) Chest X-ray showing mild improvement of left upper lobe consolidation.

On day 2 of hospitalization, his hemoptysis and breathing deteriorated, and his white cell count rose further to 18.1 K/uL. His supplemental oxygen requirements increased, and chest X-ray (CXR) (Figure 2(a)) showed worsening left-sided infiltrates. Following this, the antibiotic regimen was broadened to cefepime 2 gm three times a day for 8 days and vancomycin dosed by the pharmacy for 5 days. Urine *Legionella* antigen, *Streptococcus pneumoniae* direct antigen, *Aspergillus*, *Blastomyces*, *Coccidiodes*, histoplasma antibodies, and repeat SARS-CoV-2 testing were all negative. Blood cultures and sputum cultures were obtained and were found to have no growth. Bronchoscopy was performed revealing DAH thought secondary to pulmonary edema. Intravenous (IV) steroids were started, and further infectious and autoimmune workup was undertaken. Bronchoalveolar lavage sampling was negative for acid-fast bacilli, *Nocardia* cultures, and CMV DNA. With the continued decline in respiratory function, he underwent endotracheal intubation.

While on mechanical ventilation, aggressive diuresis was initiated for pulmonary edema. Thereafter, he was weaned off the ventilator and extubated within a week. His hemoptysis resolved, but he continued to cough with infrequent mucoid phlegm. Antibiotics were discontinued after ten days when he was clinically better, and repeat CXR exhibited slight improvement in left-sided infiltrates along with the resolution of his right lung consolidation.

Steroids were transitioned from IV to oral prednisolone 40 mg daily for 26 days followed by a taper to cover for suspected alcoholic hepatitis. His mental function improved with alcohol withdrawal treatment and he became more alert and oriented. On further questioning, he recalled having exposure to sick kittens and mice few days before admission. He was tested for Hantavirus, Q fever, leptospirosis, and toxoplasmosis, and empiric treatment with doxycycline 100 mg twice a day was initiated for 3 weeks. TB testing (QuantiFERON, sputum acid-fast bacilli, and PPD) and antibodies for leptospirosis and Q fever were all negative.

On day 18, the patient was hemodynamically stable with complete resolution of respiratory failure and was deemed stable for discharge. Four days after discharge, Hantavirus IgM returned positive with equivocal IgG. Retrospectively, his presentation correlated with HCPS. Upon follow-up in the clinic 10 days after discharge from the hospital, he reported improvement in cough and dyspnea. Outpatient CXR (Figure 2(b)) revealed mild improvement of left upper lobe consolidation.

## 3. Discussion

**Figure 3:**
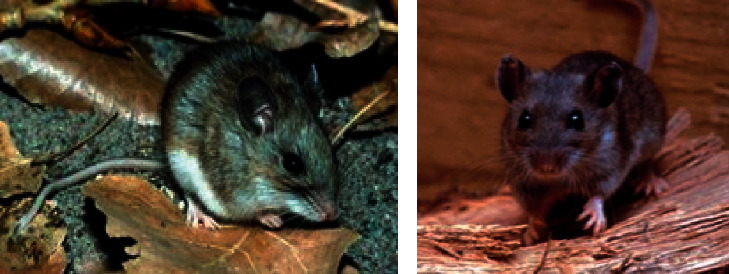
(a) Deer mouse [6]. (b) White-footed mouse [6].

Hantaviruses are enveloped, segmented negative-strand RNA viruses that belong to the Bunyaviridae family. Human spread occurs by inhalation of aerosolized excreta from infected rodents [4]. In the US, rodents that carry hantavirus include cotton rat, deer mouse, rice rat, and white-footed mouse (Figure 3) (transmission in the Midwest is predominantly from deer mouse and white-footed mouse). Sin Nombre virus (SNV) is most prevalent in North America, while in Central and South America, the most common one is Andes (AND) virus. Person-to-person transmission has not been documented in North America, Europe, or Asia [5].

Close to 40 different species of Hantaviruses have been identified, and 22 are considered to cause infections in humans [7]. The hantaviruses that circulate in Europe and Asia called the “old world Hantaviruses” cause HFRS which is characterized by hemorrhagic manifestations such as skin petechiae and ecchymoses, epistaxis, hematuria, hematemesis, melena, fatal intracranial hemorrhages, and renal failure [8]. Hantaviruses in the Americas called the “new world Hantaviruses” cause the HCPS [5, 8]. HCPS was first described in the US in 1993. SNV is the most common cause of HCPS in North America [8]. HCPS has been listed as a notifiable disease since 1995 in the US. From 1993 to 2017, a total of 728 cases of HCPS have been reported in the US (17 in South Dakota) [9, 10].

### 3.1. Hantavirus Cardiopulmonary Syndrome

HCPS has a severe disease course with a high case fatality rate ranging from 30 to 50%. The disease course progresses through 3 distinct phases: prodromal, cardiopulmonary, and convalescent/recovery [8]. The prodromal phase is characterized by nonspecific complaints including fevers, headaches, malaise, myalgias, nausea, vomiting, and abdominal pain lasting anywhere from 1 to 5 days like our patient's presentation. There are usually no upper respiratory tract symptoms in this phase. Another characteristic feature of the prodrome is thrombocytopenia. About 80 to 95% of people will have a platelet count of less than 150,000 units [4].

The cardiopulmonary phase consists of cough, dyspnea, and hypoxia with the development of noncardiogenic pulmonary edema from increased permeability of pulmonary capillaries referred to as “pulmonary leak.” In severe cases, it progresses to myocardial dysfunction resulting in cardiogenic shock. Complete blood count and a peripheral smear aid in the diagnosis of HCPS. Thrombocytopenia (platelets <150,000 units), hematocrit >50% in men and >48% in women, lack of toxic granulations in polymorphs, left shift of myeloid series, and >10% immunoblasts are some typical features of HCPS. The presence of four out of the abovementioned five findings has a 96% sensitivity for Hantavirus infection. Other laboratory findings include high levels of CK, LDH, and transaminases along with low albumin [4]. Our patient had similar findings on testing. Chest radiograph often shows bilateral interstitial markings, and some patients may have unilateral opacities in the beginning, later progressing to bilateral infiltrates [4, 5]. This phase usually lasts about a week, with a subsequent diuretic period and resolving pulmonary edema. Convalescence may take up to 6 months [8].

The definitive diagnosis is always based on serologies, the presence of IgM and IgG antibodies in the serum. The enzyme-linked immunosorbent assay (ELISA) provided by the CDC and the strip provided by TriCore Reference Laboratories at Albuquerque, New Mexico, are commonly used assays [4]. Reverse transcription-polymerase chain reaction (RT-PCR) is another method that detects viral RNA during the viremic phase of infection [4, 5].

Currently, there are no FDA-approved treatments for Hantavirus diseases except for supportive management for respiratory failure and cardiogenic shock [11]. People are usually treated with broad-spectrum antibiotics while awaiting results like in our case. Administration of fluids should be carried out very cautiously, and if suspicion for HCPS is high, patients should be transferred to the Intensive Care Unit (ICU) early in the course [12]. Some antiviral medications studied in animal models such as ribavirin and favipiravir show effectiveness only when administered in the prodromal phase before the onset of viremia [11]. Patients with HCPS who progress to severe cardiopulmonary failure and refractory shock have very high mortality, and early extracorporeal membrane oxygen (ECMO) has been suggested to improve outcomes in such patients [13]. However, according to a case report, a good response to continuous high-volume hemofiltration for a limited time duration may assist in identifying patients who may improve with conventional ICU management decreasing the need for ECMO and transfer to ECMO-capable facilities [4].

## 4. Conclusions

HCPS has high mortality, and no antiviral treatments or vaccines have proven to be efficacious to this date. It can present with DAH, and an extensive negative infectious workup in the case of pulmonary infiltrates should raise the suspicion for this diagnosis, especially if exposure to rodents has been documented. Our patient had multiple tests conducted for COVID-19 which were negative. Not all unexplained cases of shortness of breath and pulmonary infiltrates should be attributed to COVID-19 during this pandemic, and effective history taking can help identify other rare causes of infection. More research needs to be undertaken to prevent the fatal outcomes of HCPS.

## Data Availability

Previously reported published data which can be found at Google Scholar and PubMed were used to support this study and are available and reported in the manuscript. These prior studies (and datasets) are cited at relevant places within the text as references. Pictures were extracted from https://www.cdc.gov/hantavirus/rodents/index.htm.
